# Mental health experiences of HIV/TB healthcare workers during the COVID-19 pandemic – lessons for provider well-being and support from a qualitative study in seven South African provinces

**DOI:** 10.1186/s12913-023-09716-w

**Published:** 2023-07-04

**Authors:** Blia Yang, Rafaela Egg, Heena Brahmbhatt, Mahlodi Matjeng, Thanduxolo Doro, Zandile Mthembu, Batanayi Muzah, Brendon Foster, Johanna Theunissen, Ashley Frost, April Peetz, Katie Reichert, Graeme Hoddinott

**Affiliations:** 1 Panagora Group, Walker Creek Office Building, 90 Florence Ribeiro Ave, Muckleneuk, Pretoria, South Africa; 2 USAID, 100 Totius St, Groenkloof, Pretoria, South Africa; 3 Desmond Tutu TB Centre, Department of Paediatrics and Child Health, Faculty of Medicine and Health Sciences, Stellenbosch University, Cape Town, South Africa

**Keywords:** COVID-19, Mental health, Healthcare workers, South Africa

## Abstract

**Background:**

COVID-19 has substantially reshaped health service delivery. Healthcare workers have had to serve more clients, work longer shifts, and operate in conditions of uncertainty. They have experienced multiple stressors related to the additional ‘labour of care’, including managing the frustration of inadequate therapeutic or symptom relief options, witnessing clients dying, and having to give this news to clients’ family members. Ongoing psychological distress among healthcare workers can severely undermine performance, decision-making and well-being. We sought to understand the impact of the COVID-19 pandemic on the mental health experiences of healthcare workers delivering HIV and TB services in South Africa.

**Methods:**

We used a pragmatic and exploratory design to understand HCWs’ mental health experiences with in-depth qualitative data. We implemented the study in ten high HIV/TB burden districts across seven of South Africa’s nine provinces among healthcare workers employed by USAID-funded implementing partners. We conducted in-depth interviews (virtual) with 92 healthcare workers across 10 cadres.

**Results:**

Healthcare workers reported experiencing a range of extreme and rapidly fluctuating emotions because of COVID-19 that negatively impacted on their well-being. Among these, many healthcare workers report experienced a great deal of guilt at their inability to continue to provide quality care to their clients. In addition, a constant and pervasive fear of contracting COVID-19. Healthcare workers’ stress coping mechanisms were limited to begin with, and often further interrupted by COVID-19 and non-pharmaceutical response measures e.g., ‘lockdowns’. Healthcare workers reported a need for greater support for managing the everyday burden of work – not only when experiencing a mental well-being ‘episode’. Further, that whenever they were exposed to stressor events, e.g., supporting a child living with HIV who reports sexual abuse to the healthcare worker, that this this would trigger additional support interventions and not rely on the healthcare worker seeking this out. Further, that supervisors spend more effort demonstrating appreciation toward staff.

**Conclusions:**

The COVID-19 epidemic has added significant mental health burden for healthcare workers in South Africa. Addressing this requires broad and cross-cutting strengthening of everyday support for healthcare workers and centring staff’s mental well-being as core to delivering quality health services.

## Background

The Coronavirus disease 2019 (COVID-19) pandemic has substantially reshaped health service delivery and the services provided by healthcare workers (HCWs). Low-and middle-income countries (LMICs) face greater challenges, as they simultaneously grapple with the effects of the COVID-19 pandemic on existing in-country epidemics. Their health systems, which were already under strain, now face additional challenges related to service access and COVID-19 mitigation and prevention [1–4].

The COVID-19 pandemic exposed the cracks in health systems globally, where bed and oxygen shortages and COVID-19 associated illness and death had a significant impact on the mental health of HCWs. While improved understanding of COVID-19 transmission, access to personal protective equipment (PPE), and the scale-up of vaccine access, (often prioritizing HCWs), has somewhat mitigated HCWs’ direct risk of COVID-19 infection, there is significant vaccine hesitancy among HCWs globally and in South Africa [5]. Due to the COVID-19 pandemic, HCWs have had to serve more clients, work longer shifts, and operate in conditions of uncertainty around physical risk [6]. They have experienced multiple stressors related to the additional ‘labour of care’ under the COVID-19 pandemic, including managing the frustration of inadequate therapeutic or symptom relief options, witnessing clients dying, and having to give this news to clients’ family members [7], in addition to human resources for health constraints, with many HCWs missing work due to COVID-19 illness and exposure. In addition to stressors in healthcare facilities, HCWs continue to experience stressors of COVID-19 associated illness and death in their families and communities as well as the economic and social impact of the COVID-19 pandemic, including the loss of employment and increased poverty, substance use, and domestic violence [8]. Several studies have reported on the negative impact of the COVID-19 pandemic on HCWs’ mental health [6–12]. Understanding the long-term effects of the COVID-19 pandemic on health systems, especially in LMICs and among HCWs delivering care, is a global health priority at this time, as well as for future emergency crisis and pandemic preparedness.

Ongoing psychological distress among HCWs can severely undermine performance, decision-making and well-being [12]. It can also lead to increased absenteeism and negatively affect productivity and team morale [13], which can in turn lead to poorer health outcomes for clients. Studies have also highlighted the need to develop targeted mental health support interventions for HCWs; understanding the mental health impact of COVID-19 on HCWs in different settings is important to have a more responsive and comprehensive strategy to meet their needs [11]. Understanding HCWs experiences and mental health support remains a high priority.

HCWs in South Africa are facing the highest number of confirmed COVID-19 cases on the African continent, with 3 424 534 confirmed cases and 90 854 COVID-19 associated deaths (by December 29, 2021) [14], although excess mortality is multi-fold in South Africa’s HIV, tuberculosis (TB) [15], and gender-based violence (GBV) epidemics [16]. The country has the world’s largest HIV epidemic, with approximately 7.8 million people living with HIV (PLHIV)—approximately 20% of the total worldwide [17]. South Africa also has one of the world’s highest TB incidence rates, at 615 per 100 000 population [18]. The country has also been battling with an increase in GBV—South Africa has the highest rates of GBV globally, including domestic violence and rape [16, 19]. During the COVID-19 pandemic, there has been an increase in femicide and GBV in South Africa [19]. The country already has one of the highest poverty and social inequity crisis globally [20]. The COVID-19 pandemic has had a significant impact on the economy in the country, with unemployment increasing from 23.3 percent at the end of June 2020 to 34.4 percent at the end of June 2021 [21]. Food insecurity has also been exacerbated during the COVID-19 pandemic. Consequently, HCWs are working within a context of multiple overlapping epidemics during the global COVID-19 pandemic, which highlights the syndemic nature of COVID-19 and the mental health impact of historical and continued trauma.

In several high HIV-burden districts in South Africa, the United States President’s Emergency Plan for AIDS Relief (PEPFAR) through the United States Agency for International Development (USAID), supports the implementation of critical healthcare through funding to local non-governmental organizations (NGOs). These NGOs provide technical support to strengthen HIV and TB health services in the public sector.

The objective of our qualitative study was to understand the impact of the COVID-19 pandemic on the mental health experiences of HCWs working for USAID implementing partners delivering HIV and TB services in South Africa. The delivery of critical care to clients with co-morbidities—such as HIV and TB—hinge on the wellbeing of frontline HCWs, who experienced unprecedented levels of mental health trauma from the direct and indirect effects of the COVID-19 pandemic. The implications of our study can help bring awareness and encourage a policy change to strengthen mental health support services for HCWs.

## Methods

### Setting

Our study was implemented in ten districts across seven of South Africa’s nine provinces. Geographical location areas were defined as urban, peri-urban, or rural. The public health services are managed by the provincial departments of health, with primary, secondary, and tertiary level facilities. USAID-supported HCWs are employed by the NGOs to provide support in high HIV/TB-burden primary and secondary level health facilities in these districts. Our study population was composed of: (1) office-based HCWs stationed at the NGOs; (2) facility-based HCWs stationed at the clinic [primary health facility] or the hospital [secondary health facility]; and (3) community-based staff working within the facility’s catchment area.

### Study design

We used a pragmatic, exploratory, and case descriptive qualitative study approach to understanding HCWs’ mental health experiences with in-depth qualitative data.

### Sampling, recruitment, and sample

The study was implemented with USAID-supported NGO HCW staff. HCWs met the eligibility criteria if they were 18 years or older. The sample size was determined by data saturation. To get diverse views and perspectives, the following ten cadres of HCWs were asked to participate: (1) linkage officers, (2) administrative clerks, (3) nurses, (4) doctors, (5) data capturers, (6) orphans and vulnerable children (OVC) officers, (7) group facilitators, (8) care workers for children/youth, (9) mentors, (10) lay counsellors. Participants were recruited by advertising the study to all NGO staff via email and through routine staff communiques. Potential participants contacted the study team, who provided information for consent and iteratively reviewed the sample profile to ensure diversity. Interviews were conducted in the participant’s preferred language. Participants were provided with information on free mental health counselling and social services support available, and a list of these services was provided to each participant in case they wanted to access support after the interview. Participants were provided with compensation for their time in the form of mobile airtime of ZAR150 (~ US $10.00), which was standardized to the local airtime provided by other studies being conducted in the area. The airtime was provided for participants to be able to reach out to seek mental health counselling or social services support, as needed. A research packet was provided to each participant, which included the participant information leaflet, consent form, and PPE (hand sanitizer and a mask). None of the HCWs were known by the interviewers prior to the study, and the interviewers and the participants did not reside in the same communities. Confidentiality was assured by using research code names for each participant instead of names. All audio and video recordings were saved on a password-protected Google Drive only accessible to the research team.

### Data collection processes

We conducted semi-structured, in-depth interviews using a virtual platform (Zoom, Google Meet, Microsoft Teams) in order to observe COVID-19 safety protocols. All interviews were audio-recorded or video-recorded with the permission of participants. At the start of the interview, we collected socio-demographic information (job title, number of years in the position, and type of employment [full- or part-time]), geographical location (urban, peri-urban, or rural), and workplace (office, health facility, community) to inform sampling choices. The interview protocol and interview guides were developed to inform our study objectives, which were: (a) how do HCWs usually manage their stress and how has this changed since the COVID-19 pandemic; (b) how has the COVID-19 pandemic affected HCWs personally and professionally; and (c) what do HCWs think they need to better manage their mental health and well-being. Each topic area included open-ended probes to facilitate detailed descriptions by participants.

### Data analysis

Qualitative data analysis was iterative and conducted alongside data collection. The audio and video recordings, along with field notes, were used to summarize detailed case descriptions within 48 h of the interview. The case descriptions were shared among the data collection team and with an expert socio-behavioural scientist to clarify participants’ meanings and inform future data collection. We also compared between cases to identify both recurrent themes common to all participants, as well as exceptional experiences. Data were organized thematically using NVivo qualitative data analysis software. Finally, we considered the influence of setting (e.g., urban vs. rural), HCW role, and participant demographics on these recurrent themes to further clarify patterns in the data. In the findings section, we present illustrative examples to demonstrate these patterns.

### Ethical considerations

The study received ethical approval from Stellenbosch University Health Research Ethics Committee (N20/11/073_COVID-19) and the provincial health research committees of each participating province/district. All participants completed informed consent forms. The participant information leaflets and consent forms were translated and made available in South Africa’s 11 official local languages.

## Findings

Our sample consisted of 92 HCWs out of 188 potential participants, from ten USAID-supported districts across seven provinces in South Africa, with data collection from March 31-June 4, 2021. Sampling continued to saturation per district. Thematic redundancy was achieved with an average of about nine interviews per district. All 10 cadres of HCWs described in the sampling participated. Interviews were typically between 60–90 min. We sampled purposively for diversity by district and HCW cadre and balanced the sample by sex to reflect the HCW population, which is skewed towards female (see Table 1). We used the subsections of the in-depth interview guide to deductively structure the four theme categories and 11 theme clusters (see Table 2).

**Table 1:** Socio-demographics of the participants

**Geographical areas of work**	**N/D (%)**
Urban	30/100 (30%)
Peri-urban	32/100 (32%)
Rural	38/100 (38%)
**Age group across provinces**	**N/D (%)**
18–25 years	11/92 (12%)
26–32 years	30/92 (33%)
33–40 years	30/92 (33%)
41–48 years	11/92 (12%)
> 48 years	10/92 (11%)
**Sex distribution across provinces**	**N/D (%)**
Female	64/92 (70%)
Male	28/92 (30%)

**Table 2:** Theme categories and clusters

**1. What is it like to be a HCW working during the COVID-19 pandemic?**
A. HCWs experience a range of extreme emotions
B. Guilt for not being able to continue providing quality care to clients
C. Constant and pervasive fear of contracting COVID-19
**2. How are HCWs coping under the COVID-19 pandemic?**
A. Coping mechanisms among HCWs
B. The intersection of culture and gender as a barrier to the uptake of mental health support
C. Resilience and adaptability of HCWs
**3. What additional support do HCWs need?**
A. Support to manage the everyday burden of work to function optimally
B. Support and referral for specific stressor events
C. Support and referral for HCWs experiencing a chronic mental health disorder
**4. What additional support do supervisors recommend?**
A. Appreciation toward staff
B. Professional mental health support

We named the first theme category, “What is it like to be a HCW working during the COVID-19 pandemic?” with the first theme cluster within this category as, “HCWs experience a range of extreme emotions.” During South Africa’s first COVID-19 lockdown, which began in March 2020, HCWs expressed feelings of uncertainty and anxiety in their personal and professional lives, reporting panic attacks, sleepless nights, and loss of appetite:

“I experienced a lot of anxiety to the point I had panic attacks, not knowing what was going to happen, the pressure to get things right without the time to figure it out.” (Female social worker, 47 years).

“I lost two of my grannies to COVID-19. One of my grannies was the breadwinner. For three days we did not have food. We were sitting together, we don’t have food, we don’t have a plan, we don’t have anything. It was the time of avocados [season]. We ate the avocados for three days. We said, ‘God, thank you.’ We managed to cope, although it was very hard.” (Male data capturer, 26 years).

HCWs expressed concern about losing their jobs if they did not reach targets, reporting that it was more difficult to reach clients during the COVID-19 pandemic.

“It becomes a challenge when people are not coming into the hospital, it makes it difficult to reach the targets the funders want. If we don’t reach the targets, obviously we won’t get funding, then that means I’ll lose my job.” (Female social worker, 24 years).

HCWs, especially those who are parents, described feeling overwhelmed by the additional duties of taking care of children at home, helping children with schoolwork, and also now working from home:

“I’m struggling a lot to take care of the children because I’m working and studying at the same time…I have no time, my life is still difficult. I’m overwhelmed.” (Female social auxiliary worker, 32 years).

HCWs also described the grief they experienced at the loss of family members, colleagues, or clients due to COVID-19. Many who lost loved ones were in shock because of how quickly COVID-19 progressed:

“This thing to me is really, really, really hurtful. This thing changed my life. One of my colleagues phoned me, said, ‘My mom has passed away.’ I couldn't even have words to say to her. It took the father, and the father’s baby and her mom, to the other side. I have lost family and friends; I’ve lost so much. I am wounded.…The last one, the funeral of her mother, I couldn’t attend it, because the pandemic was still strong and hitting.” (Male social auxiliary worker, 32 years).

In addition, participants shared grief at the loss of clients due to COVID-19 and needing additional support for emergency response services:

“Something bad happened [starts crying], one of my beneficiaries [clients] passed away, the child was calling me, she was staying far from my home, she was saying, ‘My mama can’t breathe. It’s only me and my mama at home.’ I couldn’t get a car to travel there, it was late. I tried calling the ambulance, they didn’t even pick up. They never picked up, then I called the police. I tried to call the police, they said they don’t have cars and they said to call the ambulance…I asked the child to ask the neighbor. I feel like I failed her, the child trusted me…The child is HIV-positive. She doesn’t have a dad. The child was nine. The mother couldn’t breathe, she passed away from COVID.” (Female youth facilitator, 43 years).

HCWs reported a continuing need to manage the ongoing effects of COVID-19 through new waves of infection; the ongoing social, economic, and health system disruptions; and their own lingering trauma. The effects of the pandemic manifested among HCWs in various ways:

“There were moments of hopelessness; no one knew what was going to happen.” (Female doctor, 34 years).

“It has made changes that we are not used to in our livelihood. It is now our new normal; the pandemic is not going away anytime soon. Others cannot work because of the pandemic, they have lost their jobs because of the pandemic, they have been retrenched…Others are saying, ‘I am living for today because I don’t know what will happen tomorrow’.” (Male social auxiliary worker, 33 years). The second theme cluster within the first theme category was “Guilt for not being able to continue providing quality care.” HCWs expressed how the COVID-19 pandemic affected the services they were providing to HIV and TB clients, OVC, and survivors of GBV. They communicated a sense of guilt for not being able to provide quality care to clients during the pandemic, which at times resulted in interruptions in treatment.

“With HIV patients, they are not accessing the health facility as before, they are not getting their full check-up, they are not getting feedback whether their viral load is suppressed. Before COVID, I would know my patients would go to the clinic, they would be monitored. It’s making our work a struggle…Your hands are tied; you can’t assist them like you used to.” (Female caregiver mentor, 38 years).

“Before, we used to help patients get their medication. It affected the relationship between me and my beneficiaries [clients], my patients. My patients miss me…They say, ‘Now it’s the pandemic, you abandon us.’ They don’t trust us now.” (Female youth facilitator, 43 years). “It has affected us hugely. Everything has been shifted to focus on COVID-19; it even becomes a norm now where no one in the community comes out to speak on HIV and TB. We are neglecting our HIV and TB patients…We are supposed to do home visits for the patients who test positive for TB. I could not get to them, they have not gone to any health facility, they are not following any regulations. If there was no COVID, I would have been there daily to help, but I wasn’t able to in the beginning because of the lockdown.” (Female caregiver mentor, 38 years).

HCWs who provided support to GBV survivors, OVC, and children living with HIV (CLHIV) shared stories of social and economic challenges that clients faced:

“Oooohhh it makes us feel [starts crying]…like we are not doing enough…There was a case of a child who committed suicide, he didn’t have enough to provide for the family. Because of the pandemic we couldn’t go check on this. It’s hurting us. It’s giving us a bad record to our community. We are failing them even though it is beyond our power. The kid was infected [with HIV]. We normally give them food parcels and stationery, but we couldn’t [due to the lockdown].” (Female youth facilitator, 33 years).

“Rape is also high in our community…the child is being raped on a daily basis, her cousin rapes her daily, because her parents died. You find it so disturbing…they are all caged in one house, one room…” (Female social auxiliary worker, 47 years).

Similarly, HCWs expressed guilt in terms of managing their teams in the clinics:

“Management was the most difficult part: I felt responsible for my team, asking them to go to the facility, putting them at risk. A lot of them got COVID.” (Female doctor, 34 years).

The third theme cluster within the first theme category was a “Constant and pervasive fear of contracting COVID-19.” HCWs stated that they were in constant fear of contracting COVID-19, re-infection, or infecting their family with COVID-19.

“They are saying you can easily get it by touching, even touching the door key. That put too much pressure and anxiety on me…I would lose my mind, the way I was afraid of it…There were sleepless nights. The other night, I had to feel my heart, it was pumping very fast. I even went to go see a doctor who said to me I must not worry about this pandemic. After I disclosed to her that my colleagues contracted it and tested positive [for COVID], I almost lost my mind…if I get this COVID then I will contract it to the whole family.” (Male social auxiliary worker, 32 years).

“Professionally, it affects us healthcare workers, especially us doctors, we are going into the hospitals as frontline healthcare workers, we go into environments where we know there are COVID cases. We have to attend to the patients. Even though you know you are protected with the masks and such, you know fully that you are at risk. Then you go home, then you know you are putting them at risk. When you are at home, you are talking to your wife and to your kids, you can’t wear a mask.” (Male doctor, 46 years).

HCWs who conducted home visits reported being at an increased risk of contracting COVID-19, as most people in the communities were not wearing masks and were not following COVID-19 regulations.

“For my beneficiaries [clients] that I follow up on, you get into a household, they are not even wearing masks, they don’t even have hand sanitizer to wash their hands or soap to wash their hands. They say, ‘we have to use the soap to wash our bodies.’ There will be five that test COVID positive that live in a one-room shack…You see for work, it’s an everyday scare. We are highly, highly at risk.” (Female caregiver mentor, 38 years). Participants also expressed concern about community misconceptions and conspiracy theories related to COVID-19, with reluctance to get vaccinated.

“In the community, they still don’t believe in COVID, it’s not like when someone dies from a knife then the community sees this. With COVID, no one can see what happened to the community member, so they don’t believe COVID exists. They are in denial.” One COVID-19 conspiracy theory is that the vaccine was designed to “kill off” the population.” (Female caregiver mentor, 38 years).

“I think it has a lot to do with poverty, I think we have been promised a lot but not given it, so they don’t believe things anymore from the government. They say COVID was brought by the government to kill them…We give them examples that ARVs [antiretrovirals] were started as a trial, polio vaccine started as a trial, they will say, ‘It’s fine, I will drink my ARVs and my child will go for immunizations, but I will not go for the COVID vaccine’.” (Female caregiver mentor, 38 years).

HCWs also shared their own COVID-19 misconceptions and vaccine hesitancy.

“For me, I don’t think I’ll be vaccinated: some people react badly to the vaccine. I have this situation with iron, so I think I’ll have a side effect. I don’t want to have it, so I just make sure I protect myself.” (Female case manager, 26 years).

The second theme category was, “How are HCWs coping under the COVID-19 pandemic?” with the first theme cluster within this category as, “Coping mechanisms among HCWs.” HCWs reported a wide array of coping mechanisms to deal with stress. Most shared that they had a strong faith in God and prayed regularly, which they felt helped them deal with the daily pressures of life and the additional stress brought on by the COVID-19 pandemic.

“I try to manage everything through prayer, that’s what makes me stronger. If it wasn’t for it, I think I’d have quit a long time ago.” (Female lay counsellor, 29 years).

Several HCWs described activities that they have put in place to de-stress, such as going to the gym, running, and playing soccer:

“I’m a soccer coach; I teach kids. It helps me to interact with them; it gives me hope.” (Male social auxiliary worker, 39 years).

It was common to hear HCWs say that they relieve stress by watching movies, the news, and sitcoms; chatting on social media; going to the movies or to dinner with friends; and spending time with family. There were a few HCWs who shared that they had substance abuse challenges, which they said were exacerbated when there was uncertainty in their life.

“I used to go to the gym. But after this recent loss, I just say, maybe I will take this whiskey and relax. You know, what breaks my heart [is] when I am in the street, then someone says, ‘I am sorry for your loss,’ or if I am at the gym, if they don’t recently know, then it brings back the pain.” (Male linkage officer, 30 years).

Some HCWs said that they had accessed mental health support, such as a psychologist who they had paid for themselves, employee wellness programs offered by their organization, or free mental health support available through a social services organization.

“Last year I met a [private] psychologist so we could go through this together. It was 100% helpful.” (Female social auxiliary worker, 32 years).

Seeking support was also done through religious organizations:

“In my church, we do go see someone for help. Especially when you feel you need to talk, then you go to the Pastor, then you talk to her, then she advises you.” (Female youth facilitator, 34 years).

Those who reported that they had a strong family support structure trusted and confided their challenges to their family members.

“When you combine family support with your internal resilience, it gives you that killer combination to approach difficult situations.” (Male nurse, 30 years).

In terms of awareness of mental health support, there was a difference between those who were office-based in comparison to those who were community-based and health facility-based, with the latter not as aware of available services.

“We talk to my colleagues for support on a WhatsApp group. I haven’t talked to someone professionally. I don’t even know where to go for this.” (Female youth facilitator, 43 years).

“I am not aware of any services provided by [organization name].” (Female youth facilitator, 27 years).

Office-based HCWs were more aware of the mental health support available and were able to share what services their organization provided.

“Our employer arranged regular group debriefing sessions. If someone needs one-on-one sessions or a session with a psychologist or psychiatrist, it can be arranged by the company…I also received individual counselling sessions and it has helped me.” (Male monitoring and evaluation officer, 36 years).

The second theme cluster within the second theme category was, “The intersection of culture and gender as a barrier to the uptake of mental health support.” Several HCWs shared that the term “mental health” did not exist in the community in which they grew up. They said that if someone wanted to seek mental health support, then that person might be perceived as being “bewitched.”

“In our community, there is no such thing as mental health and depression; there is only witchcraft and drugs…If someone has a mental health breakdown, they will say, ‘That person is bewitched’.” (Female caregiver mentor, 38 years).

“There is stigma to seek mental health support. They will say, ‘This person is bewitched.’ Really, this person needs access to a counsellor or a psychologist to talk…It’s like when HIV started, people had so many stories, but then people had so many campaigns, so much education, so then people had to open up their minds and those myths can go away.” (Male social auxiliary worker, 33 years).

Some female HCWs also described the perception that as a female in their culture, seeking mental health support was unfamiliar: they were taught to deal with challenges in a certain way, and seeking mental health support was not part of the conversation.

“I am black, so being black, it’s very difficult to complain about mental health issues…A black woman, you are regarded as a strong person; you were raised to withstand a lot, you find it hard to verbalize your feelings. With panic attacks, they take it lightly in the black community. It’s not simple.” (Female nurse, 34 years).

Some male HCWs shared that they were taught not to seek mental health support.

“It [seeking mental health support] can be influenced by your upbringing. A man doesn’t cry. It doesn’t mean a man literally doesn’t cry; it means a man doesn’t rush out and cry for help. You try to help yourself first, and then you look for help.” (Male nurse, 28 years).

“Mostly for a black guy, to be going around, seeking help from a psychiatrist or a therapist, that is a sign of weakness. It’s mainly stigma, most especially guys don’t want to appear to be weak.” (Male linkage officer, 29 years).

Discussions also revealed accessing African traditional health practices.

“As Africans, we believe in traditional medicine. On my third month, I make sure I come back with herbs we use through washing. It helps me to feel I know I’m protected because I use this kind of medication.” (Male administration clerk, 40 years).

The third theme cluster within the second theme category was, “Resilience and adaptability of HCWs.” Despite experiencing personal and professional hardships during the COVID-19 pandemic, HCWs expressed resilience and reported that they were adapting to the “new normal.” The “new normal” described by HCWs included normalization of adherence to COVID-19 regulations (wearing masks, hand sanitizing before entering public places, social distancing, and refraining from group gatherings).

“We just told ourselves our life is in our hands; how we react to COVID is our business. If you want to stay safe, you need to adhere to the regulations.” (Female social auxiliary worker, 29 years).

HCWs adapted professionally to the COVID-19 pandemic by developing new interventions to reach their clients:

“It was a big scramble to get the numbers of the beneficiaries [clients]. Prior to COVID, we didn’t collect children’s contact details, because we would see [the] children in person. We used to visit the communities, and now we have to phone.” (Female social worker supervisor, 47 years).

“COVID-19 impacted the management of patients: we were doing a lot of MMD [multi-month dispensing for clients on antiretroviral therapy], we stretched their treatment to try to avoid people coming back.” (Female doctor, 34 years).

The third theme category was, “What additional support do HCWs need?” with the first theme cluster as, “Support to manage the everyday burden of work to function optimally.” HCWs expressed the need for mental health support in the form of debriefing sessions with colleagues to share their professional and personal challenges.

“I think debriefing sessions can help my colleagues and I, like a support group…We don’t have that now. Maybe my colleagues have something to share that they are afraid to talk about. Expressing your feelings is very important.” (Female youth facilitator, 35 years).

“Debriefing would be the best. It’s not easy for us; we are burdened from each and every household we visit. Remember that what I hear from you in a household, it will also affect me, so we might get burned out.” (Male social auxiliary worker supervisor, 32 years).

HCWs also shared that they preferred to have an external facilitator assist with the debriefing sessions in order to “speak freely” and “not be judged.”

“If ever there is an external person [to facilitate], the people are going to open more. If it is someone within the group, then we may hear ‘so and so said this,’ even if they weren’t in the debriefing session. There may be lack of trust. We may be afraid of being judged…Hence, I say that it’s better that an external person comes in, so that there is a level of trust, to someone you don’t know.” (Male social auxiliary worker, 33 years).

Participants emphasised the importance of being trained on mental health support available to them and how best to pass similar information on to their clients:

“We need mental health training. This will help us so when we go out into the community to talk about mental health, then we can be well informed.” (Female caregiver mentor, 38 years).

More broadly, many participants HCWs said that their ability to manage the mental health stresses of their work would be increased if they were more frequently shown appreciation for their efforts:

“We could die in the line of duty. The employers should recognize the work we do on the ground in terms of remuneration, because we’re going the extra mile. But even an email to thank us would do, a personal email.” (Male monitoring and evaluation coordinator, 40 years).

“We are not affirmed…just to give us morale, just to keep going…when we are doing good, no one is there to tell us that we are doing a good job. A lot of times, we are like zombies at home, we are just coming and going to work.” (Female caregiver mentor, 38 years).

Several HCWs in rural areas shared that they had a shortage of PPE and often had to purchase their own materials for use at work.

“I have run out of PPE. I use my own and I also buy myself sanitizer. Actually, I’ve asked my supervisor [if there] are there any gloves or masks and sanitizers, so she said, ‘No.’ So I decided to buy my own so I can be protected.” (Female youth facilitator, 35 years).

“We didn’t have enough masks, especially at the peak. When we were supposed to go for home visits or the provision of food parcels [deliver food parcels for OVC, CLHIV, GBV survivors], we ran out of masks. We ran out of masks, in fact the whole PPE equipment.” (Female youth facilitator, 33 years).

The second theme cluster within the third theme category was, “Support and referral for specific stressor events.” For specific stressor events, such as the death of a colleague or someone close to the HCW, participants noted the need to have the option to share their grief through a one-on-one counselling session.

“I’ve also had relatives that have passed away…Some are very close workmates that I have worked with…Professor [name of the professor], a well-known researcher in HIV and TB, died of COVID. Another one of my mentors, also a doctor, passed away from COVID. Some are also relatives…my supervisor, the district technical advisor for Obs & Gynae [obstetrics and gynaecology] also passed away from COVID…In [names the organization], we have lost quite a number of colleagues. Because of this COVID restriction, the only thing you get is a message…it’s not the same as someone who says, ‘Sorry, sorry’ and pats your back. There is a human element missing. I feel one-on-one sessions, they are the best. When someone is next to you, you can debrief.” (Male doctor, 46 years).

“There is a colleague at work who tested COVID positive earlier this week. There is another colleague who tested COVID positive in early January. Then there is another one that tested COVID positive, and she died at work in the clinic. She couldn’t breathe, she suddenly couldn’t breathe; she was put on oxygen. By the time the ambulance got there, she was not breathing anymore. No one even stopped us to talk about this, it was business as usual. At least someone should talk to us.” (Male linkage officer, 29 years).

“I recommend someone to come and debrief [with us]…we do identify the problems of the children, the child comes to you that her uncle raped her. You are not supposed to tell anyone about this, so you keep it inside.” (Female care worker for children and youth, 34 years).

The third theme cluster within the third theme category was, “Support and referral for HCWs experiencing a chronic mental health disorder.” In instances where HCWs may need support beyond debriefing sessions and a once-off counselling session, participants expressed the need to know more about mental health, what support is available, and how to access it. The HCWs suggested having information about mental health support widely circulated in their organizations so that they would know who to contact.

“People with bipolar or with depression, they will say this person is crazy, hence if information was given on leaflets and campaigns were done so people can understand.” (Male social auxiliary worker, 33 years).

“There can be a hotline, just like COVID. It was the best way. Word of mouth, through our colleagues and social media, will also help [circulate information on mental health].” A youth facilitator (female; 43 years) suggested, “The information [on mental health] can be on the company internet. We can also have pamphlets and leaflets, because everyone may not have access to internet all the time.” (Female caregiver mentor, 38 years).

The fourth theme category was, “What additional support do supervisors recommend?” with the first theme cluster as, “Appreciation toward staff.” Several supervisors were unaware of the mental well-being of the staff they oversee and recommended showing more appreciation to their staff. They noted that this can be improved, as almost everyone was overburdened with trying to reach targets. However, there were supervisors who were aware of the need to recognize staff for their work performance.

“Me, being the type of manager I am, I’m the one who picks them up. I appreciate the effort they put in. I acknowledge when we face difficulties when we’re not feeling okay. It opens our minds to look for assistance.” (Male nurse, 30 years).

The second theme cluster within the fourth theme category was, “Professional mental health support.” Some supervisors recognized that their staff needed mental health support, as they were dealing with challenging cases in the community [CLHIV, OVC, survivors of GBV] and needed a space to debrief and reflect on their work.

“I feel I’m not supporting them enough, because I also have my targets to reach. The staff who are working in the community would benefit from a psychologist, to see them once a month. It would take a load off my shoulder. I provide them with supervision support and the therapist would provide them with emotional support; that will help.” (Female case coordinator, 24 years).

“The caregivers need support in terms of counselling, since they’re working with the clients…Working within the community, checking on beneficiaries [clients] is stressful.” (Female caregiver mentor supervisor, 34 years).

## Discussion

Our study highlighted the tremendous stress and trauma experienced by HCWs during the COVID-19 pandemic in South Africa and the need for comprehensive services to address the mental health impact. Pervasive throughout HCWs’ lived experiences was a commitment to continue to provide care to HIV and TB clients, despite the challenges brought on by the COVID-19 pandemic. HCWs felt a tremendous amount of guilt for not being able to provide quality care during this time, as they have played an active role in the advocacy of improved HIV and TB care in South Africa since the start of these epidemics. The country has a complex health system, with apartheid ending just 27 years ago, and a population burdened by social inequity and the ongoing HIV/TB epidemics, with insufficient human resources for health [22]. It was anticipated that the COVID-19 pandemic could add critical strain to the already overburdened healthcare system due to the HIV/TB epidemics in South Africa [23]. HCWs, particularly those working in clinics, hospitals, and communities, had to quickly innovate strategies to sustain HIV and TB care throughout three COVID-19 peaks. On February 17, 2021, after two COVID-19 peaks—and 11 months after the first case in the country—COVID-19 vaccines were rolled out in South Africa, with HCWs at the forefront to receive them [24].

Several studies have reported on the substantial negative impact of the COVID-19 pandemic on HCWs’ mental health [6–12]. Our findings resonate with these studies, similarities being HCWs experiencing a wide range of extreme emotions, guilt over quality of care to clients, and fear of COVID-19 infection. However, meeting HCWs’ mental health needs remains challenging. A study conducted in a hospital in Hunan province, China, revealed that even when HCWs showed signs of psychological distress, they were reluctant to participate in the support interventions, as their needs required more PPE and the restructuring of shifts to include more breaks [25]. Similarly, in our study, HCWs shared their need for resources beyond mental health support, such as PPE and vehicles to conduct home visits to clients in rural areas, in addition to more information on COVID-19 and the vaccine to dispel misinformation.

HCWs expressed a pervasive fear of contracting COVID-19 and then infecting their families. Similarly, a survey on the burden of COVID-19 on HCWs in South Africa showed that the level of concern for personal and family well-being and passing COVID-19 infection to family members was a significant concern, and that severe psychological distress was expressed by all HCW cadres who participated in the survey [26]. This has been widely reported in the literature, as HCWs are one of the groups most at risk of COVID-19 infection [27, 28], and higher COVID-19 exposure plays a significant role in psychological distress [6, 29]. HCWs reported employing a wide array of coping mechanisms to deal with this stress. A majority of participants relied on religious support systems—a finding which was unique compared to the other studies reviewed. Several HCWs mentioned physical exercise, such as soccer or running, and many reported relieving stress by watching movies or television, chatting on social media, and spending time with family and friends. HCWs who provided support to COVID-19 patients in Hubei, China, utilized self-management strategies to cope and identified sources of social support, which is similar to our study, with social support highlighted as an important coping mechanism [7]. HCWs also shared that they had accessed professional mental health support, such as a psychologist, an employee wellness program, or free assistance through a social services organization.

The critical role of management and leadership in promoting mental health support was apparent throughout the discussions with HCWs. A scoping review conducted in South Africa to understand the psychological impact of the COVID-19 pandemic on HCWs highlighted a significant experience of depression, anxiety, and post-traumatic stress among HCWs, and suggested interventions at management level and psychological support for HCWs [6]. Several HCWs shared that they would like management to affirm them for their work, and that this would show HCWs that they are appreciated. Supervisors recognized this need and acknowledged that HCWs are working under immense pressure to achieve HIV and TB program targets. Showing appreciation and expressing gratitude for HCWs has been highlighted in other studies as a means of moral support [30].

Despite experiencing personal and professional hardships during the COVID-19 pandemic, HCWs expressed resilience and reported adapting to a “new normal” with the pandemic, consistent with other studies reviewed [7, 9, 11]. HCWs shared that the increased knowledge on COVID-19 and experiences in dealing with the disease helped them adapt to the pandemic, similar to a recent study in Iran, where HCWs shared that over time they adapted to the pandemic and the new environment was normalized, with HCWs accepting COVID-19 regulations and complying with the measures put in place to protect them and clients [11].

Our findings suggest that the lack of knowledge about mental health services available (especially among community-based and health facility-based HCWs), the lack of literacy on the topic of mental health, and culture and gender biases regarding service utilization are barriers to service access and uptake. While we found that mental health support is available to HCWs, either through their employers or in the public sector, there is a critical need to create an enabling environment for service uptake. Strengthening the integration of mental health support activities into existing operations, such as staff orientation and routine meetings, may help to normalize the topic of mental health. To help normalize the topic of mental health, dialogues could be held on the various types of mental health support—support to manage the everyday burden of work to function optimally, support for stressor events, and support for chronic mental health disorders—as well as on the intersection of culture, gender, and mental health. These potentially sensitive topics can be integrated into standard in-service training sessions that allow for a safe space and open dialogue. Debriefing sessions for HCWs to share their professional experiences can be integrated into routine staff meetings, particularly to support HCWs that reported vicarious experiences of trauma through the experiences of their clients or being exposed to contextual stressors. Ensuring that trauma management support referral systems are in place for HCWs who have more serious cases of trauma is also an important element of the continuation of care for mental health support.

These recommendations are in line with other studies, which also suggest safe spaces to share challenges and contain stress and share information about mental health and services available for referral pathways [6]. As HCWs in South Africa are grappling with the COVID-19 pandemic alongside the ongoing HIV/TB epidemics, it is important that an enabling environment is created for mental health support uptake to address HCWs’ mental health needs and support them to perform optimally.

To our knowledge, this is the first study reporting on the in-depth qualitative mental health experiences of HCWs during the COVID-19 pandemic in the high HIV/TB-burden setting of South Africa. A strength of the study is its purposive sampling for diversity in HCW cadres and across ten districts in seven provinces. Another strength is the in-depth data collection that allowed HCWs to contextualize their mental health support needs relative to their lived experiences. Finally, the process of iterative analysis within a multi-stakeholder team and a consistent manner through systematizing of findings contributes to trustworthiness.

Limitations from the findings are for two reasons. First, only HCWs who were employed by NGOs who receive funding from USAID were included. Their experiences may be somewhat different from HCWs employed by government departments of health, although the facilities represented diverse geographical and social environments in South Africa. Second, the sample included only participants who volunteered to report on their mental health experiences, which may under-represent the experiences of people with the worst mental health issues who may be inhibited from speaking about these experiences in a research context. Conversely, it may over-represent the experiences of people with work-related stress who may want to discuss their concerns about work-related issues more freely. We mitigated this second potential limitation by: (1) clearly articulating during the recruitment process why we needed a variety of perspectives; (2) sampling for diversity in age, gender, district, and HCW role to reflect the healthcare workforce in South Africa; (3) collecting in-depth data and contextualizing them relative to the participants’ work and personal experiences; (4) including supervisors’ perspectives about staff needs; and (5) implementing a multi-layered analytic process to sense-check our interpretations.

## Data Availability

The qualitative data cannot be made available publicly without risking breaking participants’ anonymity. Even when personal identifiers are removed from the data, it is still possible to guess the identity of participants just by their responses. E.g., if a participant says: “in my district, where I manage X component of the health services”. This is especially important in this project where the participants are employees of USAID-funded implementing partners reporting on their experiences working in the times of COVID-19. The data are stored securely on protected servers and any requests to review these can be made to the corresponding author and Stellenbosch University Health Research Ethics Committee.
